# Sudden death in epilepsy: There is room for intracranial pressure

**DOI:** 10.1002/brb3.1838

**Published:** 2020-09-19

**Authors:** Maxine Dibué, Jochem K. H. Spoor, Marjolein Dremmen, Christiane Freiin von Saß, Daniel Hänggi, Hans‐Jakob Steiger, Philippe Ryvlin, Marcel A. Kamp

**Affiliations:** ^1^ Department of Neurosurgery Medical Faculty Heinrich‐Heine‐University Düsseldorf Germany; ^2^ Department of Neurosurgery Erasmus MC University Medical Center Rotterdam Rotterdam The Netherlands; ^3^ Department of Radiology Erasmus MC University Medical Center Rotterdam Rotterdam The Netherlands; ^4^ Department of Clinical Neurosciences Centre Hospitalo‐Universitaire Vaudois and University of Lausanne Lausanne Switzerland

**Keywords:** excitotoxicity, neuroscience, neurosurgery

## Abstract

**Introduction:**

Sudden unexpected death in patients with epilepsy (SUDEP) remains a poorly understood entity, and it is unclear whether the same pathomechanisms underlie all sudden deaths occurring in patients with epilepsy. One aspect not included in current models of SUDEP is the role of increased intracranial pressure (ICP) which can be observed immediately upon seizure activity in neurosurgical practice.

**Methods:**

We conducted a systematic review of the occurrence of edema in patients with epilepsy reported to have died of sudden death who underwent brain autopsy or postmortem brain imaging and discuss how increased ICP may contribute to clinical features of SUDEP.

**Results:**

19 eligible studies comprising a total of 623 patients were identified. Edema—mostly mild or moderate—was reported in 17% of cases and 74% of studies. 1% (*n* = 6) of the overall cases were clearly identified as having Dravet syndrome or an SCN1A mutation. In these patients, edema was found in 4 (67%) of cases.

**Conclusion:**

Edema is regularly found in patients with epilepsy classified to have died from SUDEP. We argue that seizures preceding SUDEP may in certain cases elicit acute edema which may represent an additional contributing factor in the cascade of events leading to sudden death of patients with epilepsy. Furthermore, we hypothesize that mild edema may especially progress to severe edema in patients with sodium channel mutations which may represent an important mechanism to investigate in the context of understanding the significantly elevated risk of SUDEP in patients with SCN1A mutations.

## Graphical abstract

Seizures may in certain cases elicit acute edema which may represent an additional contributing factor in the cascade of events leading to SUDEP. We hypothesize that mild edema may especially progress to severe edema in certain patients e.g. those with sodium channel mutations which may represent an important mechanism to investigate in the context of understanding the significantly elevated risk of SUDEP in patients with SCN1A mutations.

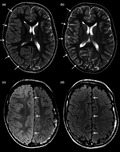

## 1 INTRODUCTION

Despite advances in understanding the pathophysiological events preceding sudden deaths in epilepsy patients and identification of certain risk factors, it remains an entity that has yet to be understood. The MORTEMUS study has been essential in shedding light on the mechanisms preceding sudden unexpected death of epileptic patients (SUDEP) establishing the understanding of SUDEP as a postictal autonomic breakdown (Ryvlin et al., 2013). However, a series of three cases of “non‐seizure‐associated SUDEP” was later published by Lhatoo et al unveiling that there are likely different types of sudden death in patients with epilepsy raising further questions (Lhatoo et al., 2016). A recent comprehensive overview summarizes risk factors and predictive biomarkers of SUDEP (Ryvlin, Rheims, & Lhatoo, 2019) and several authors have discussed potential mechanisms thereof during the recent 2 years (Allen, Harper, Lhatoo, Lemieux, & Diehl, 2019; Barot & Nei, 2019; Buchanan, 2019; DeGiorgio, Curtis, Hertling, & Moseley, 2019; Vilella et al., 2019) Insight into the likely multi‐factorial mechanisms leading to SUDEP is of the utmost importance in the journey toward preventive and interventional strategies. One aspect that is generally not included into current models of SUDEP pathomechanisms is the role of increased intracranial pressure (ICP), which can be immediately observed upon seizure activity in neurosurgical practice, and if severe can lead to a Cushing reflex exhibiting similar clinical features as observed in patients with SUDEP in the MORTEMUS study.

In the recent comprehensive SUDEP review, the authors discuss our recently published case‐report on a 21‐year‐old man with an SCN1A mutation who died 3 hr after a seizure due to transtentorial herniation, in which we discuss potential molecular mechanisms of sodium channel dysfunction promoting postictal cytotoxic edema. Isolated observations suggest that SCN1A patients may be at a higher risk for developing postictal edema due to dysregulation of sodium influx through mutated Na_v_1.1 channels into neurons and we wonder whether increased ICP may generally represent one of the early pathomechanisms contributing to SUDEP in this patient group and potentially in others also at higher risk (Büren et al., 2018; Le Gal, Korff, & Monso‐Hinard, 2010; Myers, McMahon, & Mandelstam, 2017).

## 2 METHODS

The present meta‐analysis was modeled after the preferred reporting items for systematic reviews and meta‐analyses (PRISMA) and adheres to a structured review protocol (Moher, Liberati, Tetzlaff, Altman, & Group P, 2009).

We systematically searched the PubMed database for publications reporting on cases of patients with epilepsy who died of sudden death and underwent brain autopsy or postmortem brain imaging. Two authors (M.A.K. and M.D.) independently searched PubMed database for the key words “SUDEP”, “epilepsy”, “Dravet”, “SCN1A” in combination with “autopsy”, “necropsy” and “post‐mortem”. Only peer‐reviewed original research published in English language was included. Upon uncertainty of inclusion of a publication, an additional author was consulted (J.K.H.S). The authors then independently excluded non‐relevant articles based on review of the full‐text articles before comparing selected articles. Eligibility criteria were then applied to the selected articles to obtain the final selection. The primary outcome measure, percentage of case displaying brain edema, brain swelling, or cerebral herniation among all examined cases was then extracted from each article.

## 3 RESULTS

**FIGURE 1:**
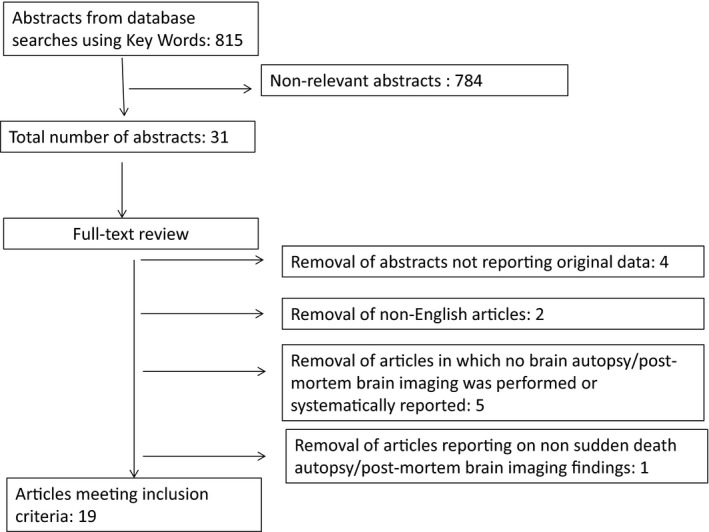
Search strategy

The described search strategy yielded 815 publications (Figure 1). Abstracts were reviewed to determine relevance which led to inclusion of 31 articles. From these articles, four were removed due to not reporting original data, two due to not being English language, one due to reporting on a case in which death was due to status epilepticus, and five were removed due to no brain autopsy/postmortem brain imaging being performed or systematically reported. The remaining 19 articles are subject of this analysis. According to the AAN level of evidence classification scheme, all studies included in this analysis must be classed as level IV evidence.

The 19 eligible studies comprise a total of 623 cases of which edema, “brain swelling” or cerebral herniation was reported in 108 cases (17%) and 74% (14/19) of studies (Table 1; Afandi, Hassanein, Roubi, & Nagelkerke, 2019; Antoniuk et al., 2001; Büren et al., 2018; Catarino, Liu, & Liagkouras, 2011; Devinsky, Ryvlin, & Friedman, 2018; Dlouhy, Ciliberto, & Cifra, 2017; Earnest, Thomas, Eden, & Hossack, 1992; Esen Melez, Arslan, Melez, Sanli, & Koc, 2017; Gronborg & Uldall, 2014; Hata, Oku, & Taneichi, 2020; Le Gal et al., 2010; Leestma, 2014; Leestma, Walczak, Hughes, Kalelkar, & Teas, 1989; Pollanen & Kodikara, 2012; Renier & Renkawek, 1990; Salmo & Connolly, 2002; Shields, Hunsaker, Hunsaker, & Parker, 2002; Thom, 2018a; Thorn, 1997). 1% (*n* = 6) of the overall cases were clearly identified as having Dravet syndrome (DS) or an SCN1A mutation. In these patients, edema was found in four (67%) of cases.

**TABLE 1:** Search results

	Publication	Patients	Total *n*	*n* of patients with edema	% of patients with edema	Additional comments
1	Verducci et al. (2019)	Patients in the North American SUDEP Registry between October 2011 and July 2018 that received a full autopsy	130	6	5%	
2	Hata et al. (2020)	Autopsy cases of two children with Dravet syndrome with novel de novo SCN1A variants dying from sudden unexpected death	2	1	50%	“advanced cerebral edema possibly associated with drowning or subsequent resuscitation”
3	Afandi & Romus (2018)	SUDEP in an adolescent after a generalized seizure	1	1	100%	“Brain tissue showed stromal oedema and dilation of the blood vessels.”
4	Büren et al. (2018)	Sudden witnessed postictal death of a young adult with Dravet syndrome and SCN1A mutation	1	1	100%	“CT revealed global cerebral edema with completely compressed ventricular cavities and no signs of traumatic brain injury, or lesions of any type.”
5	Esen Melez et al. (2017)	Cases of patients determined to have died of SUDEP (*n* = 40) out of all patients with a prior diagnosis of epilepsy referred to The Ministry of Justice Council of Forensic Medicine in Istanbul between 2007 and 2011 (*n* = 112).	40	24	60%	“All SUDEP patients having brain edema (*n* = 24, 60%) also had pulmonary edema”
6	Dlouhy et al., 2017	Sudden death at night in a child with complex febrile GTCSs	1	1	100%	“Neuropathological examination of the brain revealed global cerebral edema and marked softening of the brain with central, uncal, and tonsillar herniation—expected findings days after an anoxic brain injury.” (patient was resuscitated and died 2 days later)
7	Thom et al. (2016)	Postmortem reports in SUDEP cases from four UK neuropathology centers.	145	41	28%	“Mild degrees of brain swelling were reported in 28% of cases; this was based on report descriptions of swelling, excessive ‘fullness’ of the brain with flattening of the gyri over the convexities an exaggerated impression of the tentorium on the unci or by assessment by the level of the Greenhall line. No significant swelling or tonsillar herniation was reported in any case.”
8	Gronborg and Uldall (2014)	SUDEP cases (*n* = 9) among the 1974 patients with childhood‐onset epilepsy at a tertiary epilepsy center in Denmark followed over a period of 9 years.	9	2	22%	“Autopsy showed moderate brain edema in patient 1 and 9”
9	Pollanen and Kodikara (2012)	SUDEP cases identified from March 2005 through May 2010 in the Provincial Forensic Pathology Unit, Toronto, Ontario, Canada.	24	0	0%	
10	Catarino et al. (2011)	SUDEP case within a retrospective genetic analysis of adult patients with Dravet syndrome at National Hospital for Neurology and Neurosurgery clinics. This individual patient was found to carry an SCN1A mutation	1	1	100%	“Swollen brain with herniation (1,300 g)”
11	Le Gal et al., 2010	A case of SUDEP in a boy with drug‐resistant Dravet syndrome and SCN1A mutation	1	1	100%	Neuropathologic examination detected […], and global brain edema without mass effect
12	Salmo and Connolly (2002)	SUDEP cases among autopsy reports (*n* = 3,103) over a 10 year period at Galway University Hospital	22	2	9%	
13	Shields et al. (2002)	SUDEP cases between 1996–2000 which underwent gross examination of the brain by either a forensic pathologist or consulting neuropathologist.	64	0	0%	
14	Antoniuk et al. (2001)	Cases recognized as SUDEP among deaths registered between Jan 1990 to July 1999 that underwent postmortem examination at the Medicolegal Institute of Curitiba – Brazil.	20	11	55%	The cause of death was attributed to cerebral edema (7 cases), pulmonary edema (8), pulmonary hemorrhage (1) or cerebral and pulmonary edema (4). Although most certainly related to the convulsive disorder, these lesions do not adequately explain death in these patients.
15	Thorn (1997)	SUDEP victims examined in coroners’ autopsies from around the United Kingdom between 1994–1996	25	0	0%	
16	Earnest et al. (1992)	“Sudden Unexplained Death Syndrome” cases identified in autopsy reports of persons with epilepsy from the coroner's offices of Denver County and four adjacent counties from January 1982 through June 1987.	44	2	4%	An additional two cases were found in cardiorespiratory arrest, were successfully resuscitated, but died in the hospital due to severe brain swelling, probably due to anoxia and brain reperfusion following cardiopulmonary resuscitation.
17	Renier and Renkawek (1990)	Autopsy of a 19‐month‐old boy with severe myoclonic epilepsy of infancy (Dravet syndrome) and sudden unexpected death.	1	0	0%	The most striking features[in autopsy] were microdysgenesis of cerebellum and cerebral cortex and threefold spinal cord channels with surrounding ectopic tissue. Hippocampus and brainstem were normal.
18	Leestma et al. (1989)	SUDEP cases undergoing examination of the brain by the Medical Examiner of Cook County (Chicago), Illinois in the year of 1983	55	14	25%	
19	Terrence et al. (1975)	Cases in which death due to epilepsy was certified in autopsy records of the Allegheny County Coroner's Office from 1969 through 1973. Only cases with a complete autopsy and in which acute myocardial infarction, recent coronary occlusion, heart disease of such severity that an arrhythmia might be a reasonable possibility, trauma and evidence of aspiration could be excluded were included.	37	0	0%	In all cases in the study there were no significant findings in the general autopsy

**FIGURE 2:**
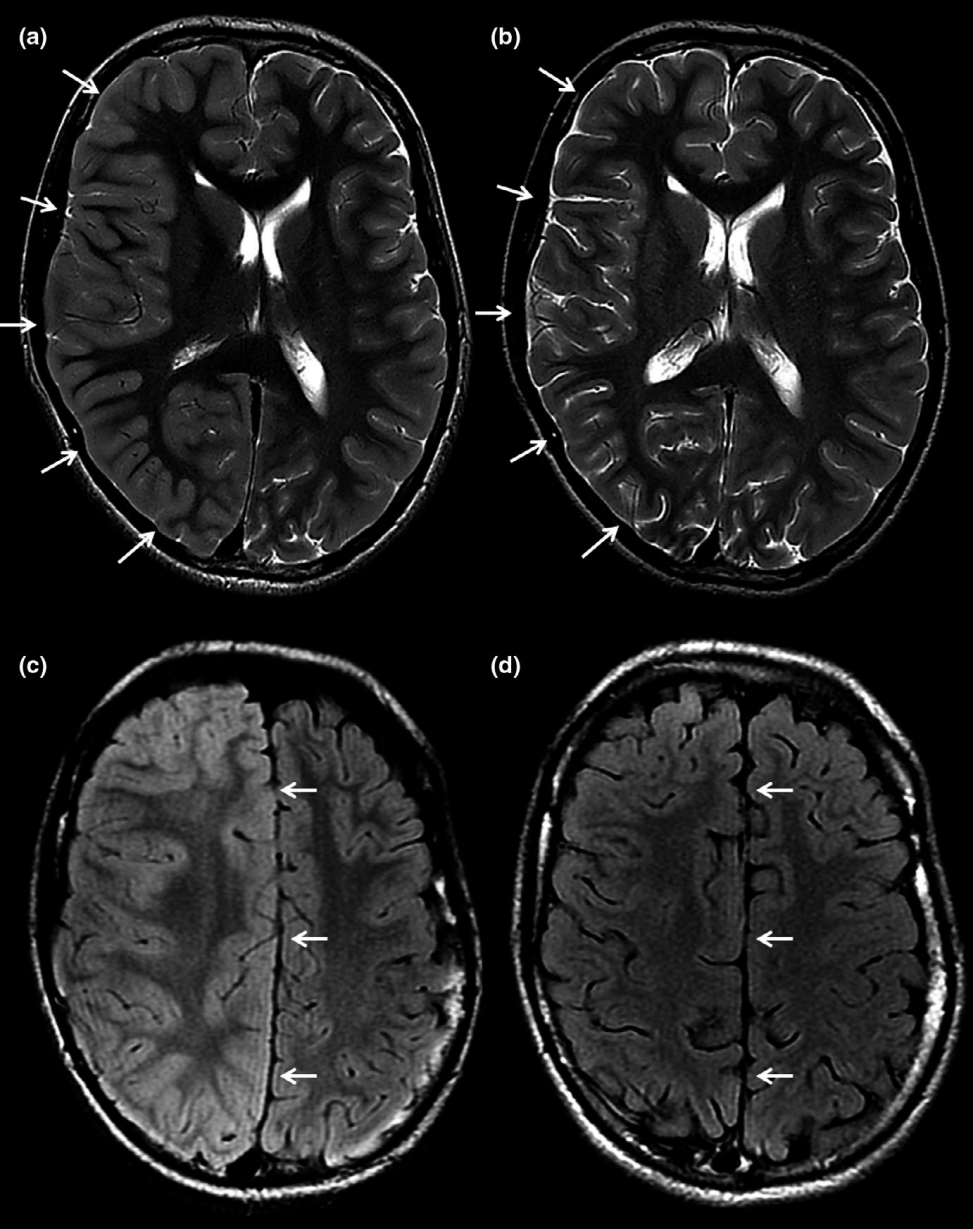
Axial T2‐weighted (a) and T2 FLAIR (b) MR images of a patient a few hours after motor seizures affecting the left side of the body and follow‐up MR images four months later (c, d). The axial images at the level of the lateral ventricles (a) and centrum semiovale (b) show diffuse swelling of the cortex of the right cerebral hemisphere with obliteration of the sulci (arrows in a) and a midline shift due to the mass effect (arrows in b). The follow‐up images at the same brain levels demonstrate normalization of the cortical swelling and midline shift (arrows in c and d)

**FIGURE 3:**
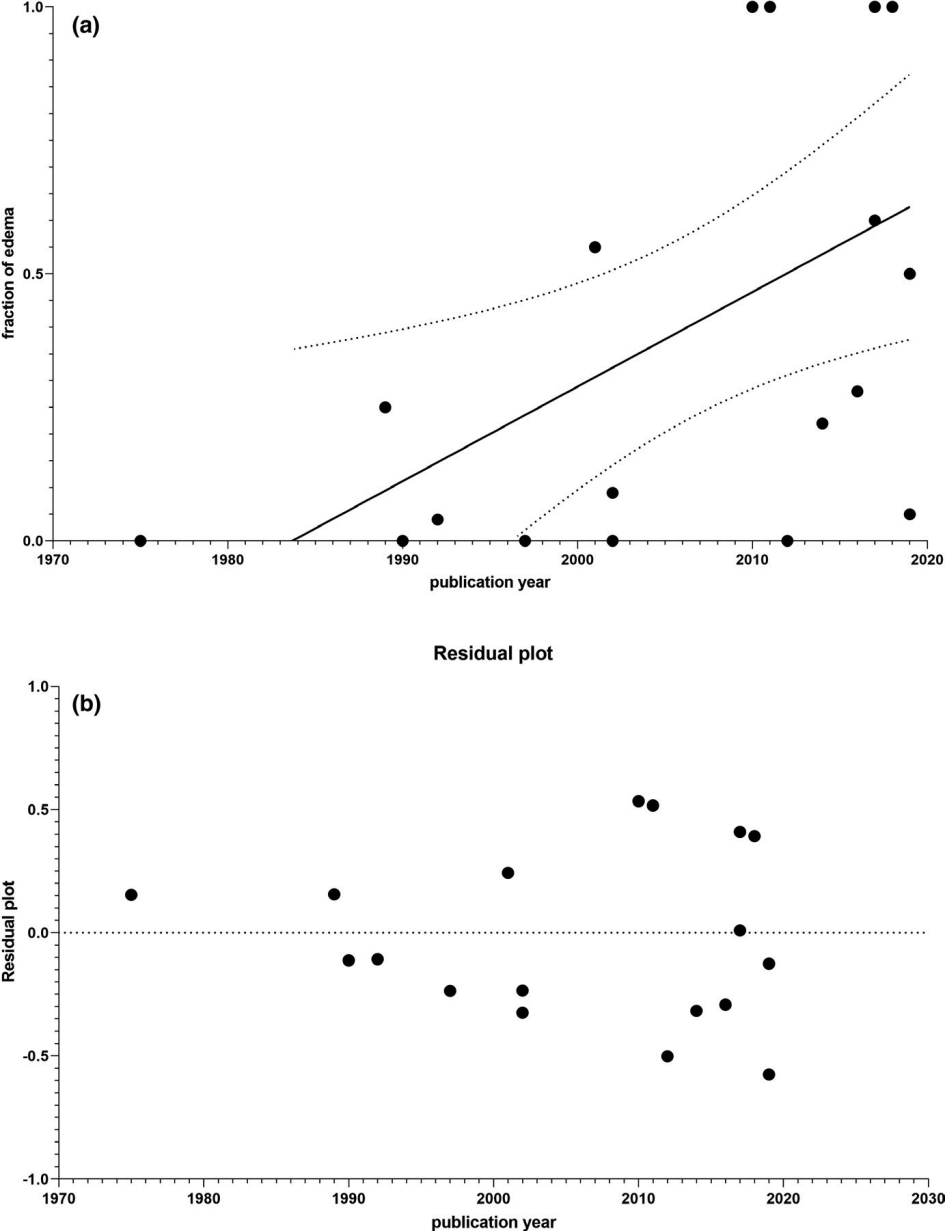
(a) Regression analysis of association of year of publication with fraction of edema. (b) Residuals of regression analysis of association of year of publication with fraction of edema

Regression analysis of association of publication year with fraction of patients with edema revealed a significantly higher rate of edema in more recent publications (Figure 3a; equation *Y* = 0,01,771*X − 35,12; *p* = .02). However, analysis of the residuals suggests that the prediction of the regression is compromised by increasing heteroscedasticity over time (Figure 3b).

## 4 DISCUSSION

### 4.1 Molecular mechanisms of ICP and ictal activity

One may hypothesize that seizures directly or indirectly promote cerebral edema: (a) Respiratory insufficiency is a basic pathophysiological mechanism of SUDEP. Respiratory insufficiency is well‐known to lead to hypoxemia and subsequently to a cerebral edema via vasogenic and/ or cytotoxic mechanisms (Sekhon, Ainslie, & Griesdale, 2017). Hypoxemia likely promotes mechanisms leading to secondary injury with various mechanisms such as microcirculatory dysfunction, arterial carbon dioxide fluctuations, or impaired cerebral autoregulation. However, cerebral edema and increased ICP directly impair cerebral perfusion pressure and cerebral oxygen delivery (CDO_2_). (b) Respiratory insufficiency will impair decarboxylation and subsequently result in an increased arterial PaCO_2_ and acidosis. The brain and its vessels are highly sensitive to the arterial PaCO_2_ and pH. Hypercapnia and acidosis are well‐known to lead to cerebral vasodilatation, increased cerebral blood volume, and subsequently to increased ICP (Lundberg, 1960). (c) Some genetic mutations (e.g., sodium channel SCN1A mutation) and conditions (e.g., TBI) promoting seizures may additionally drive cerebral edema development (Büren et al., 2018; Le Gal et al., 2010). (d) Few studies suggest that some seizure types (under certain conditions) may lead to increased ICP: During awake surgery, seizures can accidently be caused by electrostimulation and it is a well‐known phenomenon in neurosurgical practice that these seizures are frequently accompanied by massive cerebral swelling. Increased ICP and brain swelling rapidly subside if the seizure is terminated by irrigation with ice water or—more rarely—by administration of barbiturates (Tuominen, Yrjana, Ukkonen, & Koivukangas, 2013). It remains unclear if cerebral edema is caused by the seizures or for instance by the respiratory insufficiency. However, intraoperative brain swelling associated with accidentally induced seizures is likely related to the seizures as drops in PaO_2_ and raises in PaCO_2_ are usually prevented in these well‐controlled cases in the OR. To our best knowledge, intraoperative recordings of PaO_2_ and PaCO_2_ during seizures in awake craniotomies have yet not been published and capno‐masks allowing high‐flow O_2_ insufflation while measuring expiratory CO_2_ levels in awake patients are not routinely used. However, these well‐controlled cases under operative conditions might show that brain swelling is likely not caused by respiratory insufficiency. Additionally, intraoperative brain swelling occurs immediately within seconds of ictal onset which implicates neurovascular coupling to the hyperexcitation as opposed to a response to respiratory insufficiency which would likely be more delayed. ICP increases during focal motor seizures were observed in a patient (Gabor, Brooks, Scobey, & Parsons, 1984) as have prolonged ICP increases upon nonconvulsive electrographic seizures lead to a prolonged ICP increase in patients suffering from traumatic brain injury (Vespa, Miller, & McArthur, 2007). The authors hypothesize that the rise of the ICP may be caused by an increase of cerebral blood flow and volume during seizures and/ or an extracellular edema caused by increased glutamate levels (Vespa et al., 2007). Additionally, increased cerebral blood flow velocity led to an increase of ICP in 12 premature newborn infants (Perlman & Volpe, 1983a, 1983b). Transient local brain swelling has also been observed in MRI studies after recent prolonged seizure activity, generalized tonic–clonic seizures, or status epilepticus (Figure 2; Briellmann, Wellard, & Jackson, 2005; Chan, Chin, & Kartha, 1996; Kim, Chung, & Yoon, 2001; Scott, Gadian, & King, 2002). These MRI findings were considered correlates of seizure‐induced cytotoxic and vasogenic edema (Briellmann et al., 2005; Chan et al., 1996; Kim et al., 2001; Scott et al., 2002).

Taken together, some clinical evidence exists to suggest that seizure‐associated ICP increase may represent a crucial pathomechanism in certain epilepsy types; however, animal studies on this topic are nearly totally lacking. One study reporting preliminary results of novel ICP measurement methods for mice in the pilocarpine model of epilepsy found that ICP behavior of the mice with chronic epilepsy presented a characteristic of dispersion in the frequency components, which may be related to a decrease in brain compliance and failure of autoregulation (Cardim, Frigieri, & Cabella, 2016). Considering the lack of animal research on this topic, a backward translation from bedside to bench may be required.

Mechanisms of cerebral hypertension have been extensively studied in various neurological diseases such as SAH and TBI: As a consequence of raised ICP, cerebral perfusion pressure (CPP) is impaired if mean arterial pressure (MAP) remains constant (CPP = MAP – ICP). MAP is often physiologically increased to maintain sufficient cerebral perfusion. Increased cardiac output results in a reflectory bradycardia. The combination of hypertension and reflectory bradycardia is known as the Cushing reflex and is frequently observed in cerebral hypertension. Autonomic dysregulation has been observed in response to highly elevated ICP such as a cardiac uncoupling (Mowery et al., 2008), autonomic dysfunction as a result of a brainstem damage (Su, Kuo, Kuo, Lai, & Chen, 2005), and changes in heart rate and pulse pressure variability (Kahraman, Dutton, & Hu, 2010). Next to cardiovascular responses, raised ICP and reduction of cerebral perfusion lead to reduced consciousness and central respiratory dysfunction which additionally aggravate preexisting respiratory insufficiency and finally lead to unconsciousness, central apnea, and central autonomic dysfunction. Increased ICP is accompanied by temporary severe decrease of cerebral perfusion and—interestingly—by a marked suppression of cortical electrical activity, for example, in SAH patients (Bederson, Germano, & Guarino, 1995; Bederson, Levy, & Ding, 1998; Kamp et al., 2014).

Considering these fundamental pathophysiological mechanisms of cerebral hypertension and herniation, some of the risk factors for SUDEP and symptoms observed before SUDEP may reflect a seizure‐related ICP increase: Arterial hypertension is a common finding in the ictal period and much more common than hypotension. Moreover, increased blood pressure is more severe in patients with seizures with impaired awareness (Hampel, Jahanbekam, Elger, & Surges, 2016; Jaychandran et al., 2016). Many patients suffering from SUDEP in the MORTEMUS study displayed pronounced ictal tachycardia followed by progressive bradycardia and apnea in the postictal period resulting in terminal apnea and finally asystole. This sequence of symptoms could also be interpreted as symptoms of the afore mentioned Cushing reflex, which are seen in the presence of brainstem compression due to massively raised ICP. Additionally, cranial hypertension and cerebral ischemia can further be associated with a suppression of cortical electric activity (Bederson et al., 1995, 1998; Kamp et al., 2014), a feature which is frequently observed in the postictal period as postictal generalized EEG suppression (PGES; Ryvlin et al., 2019). Other features and risk factors of SUDEP may be interpreted in the view of cerebral hypertension, as for example, increased volume of the right anterior mesial temporal region is an MRI‐morphological risk factor for SUDEP but also for uncal cerebral herniation (Wandschneider, Koepp, & Scott, 2015).

## 5 SCN1A MUTATIONS IN POSTICTAL CEREBRAL EDEMA AND SUDDEN DEATH

The exceptionally high rate of SUDEP in patients with DS has spurred in‐depth research into potential contributing pathomechanisms in the past decade. A large portion of this research focusses on the impact of SCN1A mutations on cardiac function, as Nav1.1. is not only expressed in CNS neurons but also in cardiac ganglia and the myocardium, which may put patients at risk for fatal cardiac arrythmias and therefore sudden [cardiac] death (Coll, Allegue, & Partemi, 2016). A study using induced pluripotent stem cell‐derived cardiac myocytes (iPSC‐CMs) from DS patients observed increased sodium current and spontaneous contraction rates in DS patient iPSC‐CMs versus controls (Frasier, Zhang, & Offord, 2018). Contrastingly, however, a recent international multicenter trial did not identify major arrhythmias in DS which could directly explain high SUDEP rates (Shmuely, Surges, & Helling, 2020). Peri‐ictal QTc‐lengthening was, however, more common in DS, which may reflect unstable repolarization and an increased propensity for arrhythmias. In this regard, it is important to distinguish between SUDEP, which in the MORTEMUS study was shown to be a centrally induced postictal phenomenon with central apnea at the beginning of cardiorespiratory collapse and sudden cardiac death (SCD) which is the result of a diseased myocardium. This topic has been recently expertly reviewed by Verrier et al. concluding that patients with epilepsy are at a 3‐fold higher risk for SCD than the general population, due to cardiotoxicity of catecholamine surges during repetitive seizures, hypoxemia, and vascular impact of anti‐epileptic drugs leading to electrical and mechanical dysfunction (Verrier, Pang, Nearing, & Schachter, 2020). In patients with DS, repolarization abnormalities—a known risk factor for SCD, may add to this risk profile, Distinguishing SCD in patients with epilepsy from SUDEP in autopsy represents a challenge and therefore one may hypothesize that the high SUDEP rate in DS to a certain extent may be due to a significant amount of these deaths actually being SCD.

However in the present analysis, we suggest that SCN1A mutations may also contribute to postictal cerebral pathomechanisms via increased ICP and edema, and therefore to the high SUDEP rate in DS. Cerebral hypoxia leads to impairment of ion hemostasis, in particular to neuronal sodium influx, and it is suggested that voltage‐gated sodium channels (VGSCs) play a crucial role in the pathophysiology of hypoxia (Fung, Inglin, & Murek, 2016; Fung, Croning, & Haddad, 1999; Plant, Marks, & Goldstein, 2016; Stys & Lopachin, 1998). It has however yet to be investigated whether SCN1A mutations in DS alter susceptibility for hypoxia and promote hypoxia‐related cerebral edema by increasing neuronal sodium ion influx. However, we argue that ictal activity itself may promote cerebral edema, as excessive influx of sodium through mutated VGSCs such as Nav1.1 may occur during the repetitive hypersynchronous glutamatergic firing of seizure activity. Postictal hypoxia, energy depletion, and lack of sufficient ATP ultimately cause failure of both the sodium–calcium exchange and sodium–potassium exchange further promoting sodium and calcium accumulation with hyperosmolar effects affecting the integrity of the blood–brain barrier (Rosenberg, 2000). Finally, influx of cations into neurons likely influences membrane potential facilitating further ion influx, and therefore, sodium influx through VGSCs during ictal activity may represent a critical pathomechanism leading to cerebral edema. One may therefore hypothesize that mutations in neuronal sodium channels like found in the majority of patients with Dravet syndrome may impact this pathomechanism by altering both availability of sodium ions and the sodium ion gradient.

### 5.1 Edema in autopsy of sudden death cases

The results of our systematic review indicate cerebral edema as a frequent (17%) finding in autopsy or postmortem imaging of patients with epilepsy dying suddenly (Antoniuk et al., 2001; Earnest et al., 1992; Esen Melez et al., 2017; Kloster & Engelskjon, 1999; Le Gal et al., 2010; Terrence, Rao, & Perper, 1981; Terrence, Wisotzkey, & Perper, 1975; Thom, Michalak, & Wright, 2016; Thom, Seetah, Sisodiya, Koepp, & Scaravilli, 2003). A recent systematic review of autopsy findings in SUDEP confirms that mild edema is found in approximately 30% of series (compared to 74% of articles in our analysis) and concludes that mild degrees of brain swelling with effacement or fullness of the gyri are not uncommon in SUDEP due to mild degrees of acute cerebral edema, but alone are insufficient for a cause of death (Thom, 2018a, 2018b; Thom, Boldrini, Bundock, Sheppard, & Devinsky, 2018). Cases of severe edema with cerebral herniation were not described in this review; however, in the context of the definition of SUDEP by Nashef et al. (1997), which excludes cases with a neuropathological cause of death, modern autopsy studies would likely rule out cases displaying severe edema with cerebral herniation found in autopsy from dying of SUDEP. Other series of typical sudden death in epilepsy (i.e., patients dying at night and found prone in their bed) do not show that a significant proportion of patients are adjudicated as non‐SUDEP due to the presence of brain herniation at autopsy, but such cases have been described (Leestma, 2014). However, it is likely that acute ictal edema recedes with death potentially contributing to underestimation of this phenomenon in autopsy and also in postictal MRI.

The present analysis is limited by the inherent property of systematic reviews of ignoring potentially important differences across studies. Different autopsy and brain imaging methods were used in the various studies (e.g., some cases underwent histopathological review, others macroscopical review, others MRI), and the definition of SUDEP has evolved and become more commonly adopted over the past three decades which impacts inclusion criteria.

Furthermore, the analysis is also limited by the low sample sizes of some of the included articles—especially case‐reports. This renders estimating an edema rate in the few Dravet syndrome/SCN1A sudden death cases unreliable. However, the finding that four out of six (67%) Dravet syndrome/SCN1A cases display edema compare to 17% in the general epilepsy population is of interest and warrants further investigation.

## 6 CONCLUSION

Immediate brain swelling is routinely observed intraoperatively during stimulation‐induced seizures and typically recedes immediately with termination of the seizure. Based on the mechanisms discussed in this paper and the not uncommon finding of mild edema found in autopsy of SUDEP cases, we argue that seizures preceding SUDEP may in certain cases elicit more persistent edema which—even if insufficient for a cause of death—may represent an additional contributing factor in the cascade of events leading to sudden death of patients with epilepsy. We hypothesize that in certain patients, for example, those with sodium channel mutations acute ictal brain swelling may not recede and mild edema may especially progress to severe edema which may represent an important mechanism to investigate in the context of understanding the significantly elevated risk of SUDEP in patients with SCN1A mutations. For this, future studies may use non‐invasive methods to measure ICP during seizures and the postictal period and then correlate these measurements with clinical parameters discussed in this paper such as respiration, heart rate, and MAP. Finally, backward translation from bedside to bench may offer additional insight into the pathophysiology of SUDEP.

## DISCLOSURES

Maxine Dibué is an employee of LivaNova PLC and holds stock options. Philippe Ryvlin has received speaker or consultant fees from UCB, GW pharmaceutical, Eisai and LivaNova. All other authors have no conflicts of interest to disclose.

## AUTHOR CONTRIBUTION

Maxine Dibué, Marcel Kamp, and Jochem SpooR performed the systematic review and wrote the manuscript. Marjolein Dremmen provided brain imaging illustrating the topic of the manuscript and advised from a radiologist point of view. Christiane Freiin von Saß provided medical writing assistance. Philippe Ryvlin, Hans‐Jakob Steiger, and Daniel Hänggi provided guidance in formulating the hypothesis, interpreting the results of the systematic review and conceptualizing the manuscript.

## ETHICAL STATEMENT

This paper does not report on any human or animal studies.

### Peer Review

The peer review history for this article is available at https://publons.com/publon/10.1002/brb3.1838.
